# Health expenditure and economic growth - a review of the literature and an analysis between the economic community for central African states (CEMAC) and selected African countries

**DOI:** 10.1186/s13561-017-0159-1

**Published:** 2017-06-07

**Authors:** Serge Mandiefe Piabuo, Julius Chupezi Tieguhong

**Affiliations:** World Agroforestry Centre (ICRAF), Yaounde, Cameroon

**Keywords:** Human capital, Health expenditure, Economic growth, Abuja declaration

## Abstract

African leaders accepted in the year 2001 through the Abuja Declaration to allocate 15% of their government expenditure on health but by 2013 only five (5) African countries achieved this target. In this paper, a comparative analysis on the impact of health expenditure between countries in the CEMAC sub-region and five other African countries that achieved the Abuja declaration is provided. Data for this study was extracted from the World Development Indicators (2016) database, panel ordinary least square (OLS), fully modified ordinary least square (FMOLS) and dynamic ordinary least square (DOLS) were used as econometric technic of analysis. Results showed that health expenditure has a positive and significant effect on economic growth in both samples. A unit change in health expenditure can potentially increase GDP per capita by 0.38 and 0.3 units for the five other African countries that achieve the Abuja target and for CEMAC countries respectively, a significant difference of 0.08 units among the two samples. In addition, a long-run relationship also exist between health expenditure and economic growth for both groups of countries. Thus African Economies are strongly advised to achieve the Abuja target especially when other socio-economic and political factors are efficient.

## Background

Endogenous growth models [1] highlights the importance of human capital on economic growth and development. Health is an important determinant of economic development; a healthy population means higher productivity, thus higher income per head [2]. The importance of human capital to economic growth cannot be over emphasized [3–5] because it serves as a catalyst to economic development. The contribution of health expenditure on economic development emanates from the health led growth hypothesis [6]. It considers health to be capital; therefore investments on health can lead to an increase in labour productivity,, thus increase in incomes and subsequent increase in the wellbeing of the population. Bloom and Canning [7] highlights that when labour is healthy, their incentive to develop new skills and knowledge is higher because they expect to enjoy long term benefits. However, when the labour force is characterized by workers with poor health, they turn to have an adverse effect on productivity; this explains the disparity in development in different regions of the world [8]. Fifty percent of divergence in economic growth between developing countries and developed countries is attributed to ill-health and low life expectancy [2].

The economic wellbeing of every population is ameliorated by change in technology and part of this change is due to advances in medical science. Newhouse [9] highlighted that change in technology is one of the major reasons for increase in health expenditure. The assertion of Newhouse [9] was empirically verified in the United States of America by Fuchs [10], whereby 85% of a sample of health economics scholars confirmed that technical change accounted for the rapid growth in health care expenditure in the country.

### Health care expenditure in Africa and rationale for this study

The importance of health as a key aspect of development and economic wellbeing of individuals and nations is increasingly being recognized in the world. This can be seen from a series of reforms taken by African countries to increase investments in health in order to meet the health Millennium Development Goals (MDGs). African leaders have expressed this trust through actions such as the 2001 Abuja Declaration on an increase in government funding for health by allocating 15% of the government budget to the health sector, the 2006 Addis-Ababa Declaration on community health in the African Region and the 2008 Ouagadougou Declaration on primary health care and health systems in Africa. The High-Level Taskforce on Innovative International Financing for Health Systems (HLTF) recommended that by 2009 low income countries should allocate at least US$ 44 per capita to deliver an essential package of health services. More than a third of African countries have not been able to meet both the Abuja target and the HLTF recommendation except for Botswana, Rwanda and Zambia. It is equally important to note that Equatorial Guinea has not been able to attain the Abuja target but has significantly high health expenditure per capita [11]. Table 1 below shows the level of attainment of these targets by African countries.

**Table 1:** Level of attainment of Abuja and HLTF targets

	GGHE/GGE more than 15%	GGHE/GGE less than 15%
Total health expenditure per capita more than US$ 44	Botswana, Rwanda, Zambia (3 countries)	Algeria, Angola, Cameroon, Cape Verde, Congo, Côte d’Ivoire, Equatorial Guinea, Gabon, Ghana, Guinea-Bissau, Lesotho, Mauritius, Namibia, Nigeria, Sao Tome and Principe, Senegal, Seychelles, South Africa, Swaziland, Uganda (20 countries)
Total health expenditure per capita less than US$ 44	Madagascar, Togo (2 countries)	Benin, Burkina Faso, Burundi, Central African Republic, Chad, Comoros, DRC, Eritrea, Ethiopia, Gambia, Guinea, Kenya, Liberia, Malawi, Mali, Mauritania, Mozambique, Niger, Sierra Leone, Tanzania (20 countries)

*GGE* General government expenditure, *GGHE* General government health expenditure

Source: [43]

Developing a sound system for financing health care is one of the key mechanisms to show the commitments and political will of leaders and their ability to translate these commitments into results. The desire to develop strong health financing systems is a common objective of all countries but the increasing cost of health care accompanied by the poor economic performance of developing countries and African economies in particular makes it difficult to meet this objective. The majority of African countries falls within the low and middle income range and they face a severe problem of scarcity of funds to provide quality health care services with the average total health expenditure in African countries being at US$ 135 per capita in 2010, which is only 4.2% of the US$ 3150 spent on health in an average high-income country [12].

Lack of investment in health and in actions to address the environmental and social determinants of health is a serious constraint to upgrading health outcomes in Africa, bearing in mind that the continent registers the bulk of global maternal and infant mortality [11], it equally registers the highest number of people with HIV/AIDS [11]. The perpetual increase in injuries and non-communicable diseases is putting many countries under the pressure of a double burden of disease [11].

Constraints of financing health care in Africa arise principally from the mechanisms and strategies employed in financing health care. More than 40% of total health expenditure is characterized by household out of-pocket payments which is a very regressive method of financing health care [11]. This is principally because reliance on this form of payment creates financial barriers to access health services and the risk of impoverishment is increased [13]. These flaws in health care financing accounts for inefficiencies and disparity in the allocation of health care services among nations and between rural and urban areas. The Abuja declaration came as a solution to this problem by setting targets for African countries; to invest at least 15% of government budget on health and to have less than 20% of the total health expenditure coming from out-of pocket spending. As of 2013, only Botswana can boast of meeting this target. The out-of-pocket spending in Botswana is only 8% of the total health expenditure against an average 50% for most low and middle income countries in Africa while Government expenditure on health stands at around US$ 446 per capita, which is higher than African Region’s upper middle income average of US$ 228 per capita and comparable to that of the upper middle income countries in the world [11].

African countries equally suffer from shortage in human resources for health (HRH). The World Health Organisation [11] reports that 36 out of the 46 countries in Sub-Saharan Africa are facing a HRH shortage crisis. According to Nabyonga et al. [14], the current shortage of health workers (physicians, nurses and midwives) in Africa is estimated to be at least 817 992. In order to address these problems, many African countries will have to increase their human health resources by at least 140% and review their institutions to train additional health workers [11]. Countries will encounter a lot of challenges in achieving this goal amongst which are; inequitable distribution of the available workforce, brain drain, low remuneration, reliance on expatriates in some countries, failure to attract and retain qualified staff especially in rural areas [11].

The relationship between public health expenditure and economic growth have been highly investigated in developing and developed countries, but these researchers do not come to a general conclusion. Hashmati [15] used the Solow growth model to investigate the relationship between health expenditure and economic growth using a sample of Organisation for Economic Co-operation and Development (OECD) countries from 1970 to 1992, he came to a conclusion that there was a positive relationship between health expenditure and economic growth. Kar and Taban [16] used co-integration method to verify the relationship between health expenditure and economic growth in Turkey; they noticed a negative relationship between health expenditure and economic growth. A similar study was carried out in Turkey by Yumuşak and Yıldırım [17] over the period 1980–2005. Using the co-integration method, it was also confirmed that there exist a negative relationship between health expenditure and economic growth. Arısoy et al. [18] used the same methodology in Turkey but over a longer time frame (1960–2005) and came to a conclusion that there is a positive relationship between health expenditure and economic growth. A similar study in Turkey by Eryiğit et al. [19] from 1950 to 2005 equally confirmed a positive relationship between health expenditure and economic growth.

By using short-run and long-run causality tests to verify the relationship between health expenditure and economic growth, researchers still find it difficult to come to a common conclusion. Cetin et al. [20] studied 15 OECD countries from 1990 to 2006 and concluded that there is no relationship between health expenditure and economic growth. Elmi and Sadeghi [21] verified this relationship using a sample of developing countries from 1990 to 2009 and made use of panel co-integration causality vector error correction model (VECM). Their results showed that there is a short-run relationship running from GDP to health expenditure and a bi-directional relationship in the long-run.

The role played by health expenditure was explored by Baldacci et al. [22], who made use of a panel of 120 developing countries between 1975 and 2000 to show that health expenditure within a particular time frame affects growth within that same time frame; lagged periods do not have any effect. His major conclusion was that, the effect of health expenditure on growth is not a stock effect but a flow.

In that same year, Bloom et al. [23] estimated a production function, which aggregates capital stock, labour and human capital (education, experience and health) to conclude that there is a positive and statistical significant effect of health expenditure on economic growth. Kwak [24], inspired by the Solow model, evaluated the impact of health expenditure on economic growth by breaking health expenditure into public and private health expenditure. Members of the Organisation for economic co-operation and Development (OECD) and developing countries make up his sample and his findings revealed that public expenditure on health had relatively higher positive effects than private health expenditure.

Comparative analysis of public and private expenditure on health was equally considered by Guissan and Arranz [25] who used least square regression and white heteroskedastic test to evaluate the impact of health expenditure on economic growth of 24 OECD countries from 1970 to 1996. The major findings of this study were that health expenditure plays an important role in enhancing the general wellbeing of individuals due to a greater share of individual consumption and overall productivity.

Empirical evidence on the relationship between health expenditure and economic growth can equally be seen in the study of Aurangzeb [26] who used an augmented Solow Growth model for Pakistan during the period 1973–2003. The Johansen co-integration technique and error correction model (ECM) were applied to show a positive and significant relationship between economic growth and health expenditure in both the short- and long-run.

David et al. [27] used a sample of 104 countries over the period 1960–1990 to examine the relationship between health and economic growth by applying non-linear two-stage least squares estimates (2SLS), and they noticed that good health had a positive and statistically significant effect on economic growth. They equally highlighted that, the life expectancy effect in growth regressions appeared to be a real labour productivity effect other than life expectancy proxied for workers experience.

Aguayo-Rico and Iris [28] used a sample of countries from four continents: 13 European countries, 12 African countries, 16 American countries, and 11 Asian countries to examine the impact of health on economic growth over the period 1970–80 and 1980–90. They made use of ordinary least square (OLS) regression and discovered that health capital had a significant effect on economic growth, especially with a variable that captures all the determinants of health. Similarly, Dreiger and Reimers [29] employed recent panel data co-integration technics using a sample of 21 OECD countries over the period 1975–2001, to investigate the relationship between health care expenditure and economic growth. They found the existence of a long-run relationship between health expenditures, GDP per capita and proxies for medical progress.

Causality and co-integration relationships between health care expenditure and economic growth in developing countries was investigated by Mila and Sadeghi from 1990 to 2009 and they found a short-run causality from GDP to health care spending but no short-run causality from health spending to economic growth. In the long-run, there is a bilateral causality and relationship between economic growth and health spending. Their findings showed that income is an important factor across developing countries in the level and growth of health care expenditure in the long-run. They equally outlined that the health-led growth hypothesis in developing countries is confirmed.

Bakare and Sanmi [30] investigated the relationship between health care expenditures and economic growth in Nigeria. They made use of the ordinary least square multiple regression as their method of analysis. Their results showed a significant and positive relationship between health care expenditure and economic growth. They recommended that Nigerian policy makers should continuously increase the percentage of budget allocated for health every year.

Ogundipe and Lawal [31] equally examined the impact of health expenditure on economic growth in Nigeria. They equally made use of the OLS. They noticed a negative effect of total health expenditure on growth which is contrary to the findings of Bakare and Sanmi [30] in Nigeria. Oni [32], equally verified the relationship between health expenditure and economic growth in Nigeria, she made use of multiple OLS regression. Her results showed that labour force productivity, total health expenditure and gross capital formation are important determinants of economic growth in Nigeria while life expectancy rate has negative impact on growth for the period covered by the study.

Mandiefe and Tieguhong [33] examined the contribution of public health investments to the economic growth of Cameroon. They employed the Vector Error Correction Model (VECM) as the econometric model used in their estimations. Annual time series data from 1988 to 2013 was used. The results of the estimations showed that public health investments contribute to the economic growth of Cameroon only in the long-run. This implies that public health investments boost economic growth in the long-run through efficient allocation of resources. Hence, they recommended that: first, the government should increase its health investment to 10 or 15% of its GDP as recommended by the African Union and WHO respectively; second, to enhance the provision of health care services by the private sector and third, to ameliorate the quality of health care services rendered by granting competitive awards to health units that render quality health care services.

From the empirical evidence above, it can be noticed that there is divergence in the effect of health expenditure on economic growth. We also notice divergence in results between developed and developing countries. These studies also show divergence in results of the direction of causality between health expenditure and economic growth in the short-run and in the long-run. All these divergence views give enough reasons to investigate the impact of health expenditure on economic growth in selected African countries. A lot of empirical studies Erdil et al. [34], Baldacci et al. [22], Bloom et al. [23] have investigated the relationship between economic growth and public health expenditure using granger causality test and tests for long-run association but no study to the best of our knowledge have investigated the relationship between health expenditure and economic development in the CEMAC sub-region.

The review above shows that there is abundant literature on the relationship between health expenditure and economic growth, this paper will contribute to literature by analysing performing a comparative analysis. This paper seeks to analyse how efficient the Abuja declaration can lead to economic growth by comparing the contribution of health care expenditure to economic growth of countries that meet the target with countries of the CEMAC sub region that do not meet the target. It is important to note that to the best of our knowledge this is the first study to empirically investigate the policy implications of the 2001 Abuja declaration in Africa. This paper will fill this gap by verifying the direction of causality between economic growth and public health expenditure and also to verify if there is any long-run association between them. In this direction, this paper attempts to respond to the following research question: what is the effect of public health expenditure on economic growth and the direction of causality between them in the short and long-run?

The rest of the paper is structured as follows. The methodology is outlined in Section II while Section III discusses the data. Section IV presents the empirical results before we conclude with Section V.

## Methods

### Theoretical framework

The mechanism through which public health investments affect economic growth and economic development is inscribed in the endogenous growth models. These models highlight the importance of human capital to economic growth. Neo-classical growth models explain economic growth based on savings and growth of population. Solow [35] highlighted that countries with higher savings will have higher per capita income every other thing being equal. In Solow’s model, the rate of savings and population are the principal determinants of per capita income across countries [15].

Buchanan developed a theoretical model in 1965, encouraging public authorities to increase public spending on health independent of demand. This theory highlights that inefficiency in the provision of health care should be observed not by lack of supply of health care services but by reduced quality such as congestion, infrastructure, unequal distribution of staff.

Numerous models were developed to incorporate the impact of human capital on economic development. Romer [36] and Barro [37] emphasized that human capital is a very important factor in boosting economic development. Indeed, the theoretical underpinnings of Barro are still very relevant in contemporary empirical human capital literature in Africa [38]. The augmented Solow model by [5], equally emphasized on the importance of human capital on economic growth. These endogenous models do not assume human capital as a constant. Rather, they are based on the ability of human capital to influence growth in the short-run and in the long-run. The theoretical model developed in this study highlights a functional relationship between economic growth and health expenditure which is one of the components of human capital.

$$ \mathrm{Economic}\ \mathrm{growth}=\mathrm{f}\left(\mathrm{health}\ \mathrm{expenditure},\ \mathrm{health}\ \mathrm{indicators}\right)+\left(\mathrm{trade},\ \mathrm{household}\ \mathrm{consumption}\right) $$

Thus the following econometric relation will be estimated in this study;

$$ \begin{array}{l} GDP\kern0.5em  per\  capit{a}_{i t}\\ {}\kern12em =\alpha +{\beta}_i health\  expenditure\  per\  capit{a}_{i t}\\ {}\kern12em +{\gamma}_i household\  consumption\  per\  capit{a}_{i t}+{\delta}_i life\  expectanc{y}_{i t}\\ {}\kern12em +{\omega}_i labour\  forc{e}_{i t}+{\varphi}_i trad{e}_{i t}+{\varepsilon}_{i t}\end{array} $$

Where i = individual country component

t = time component from 1995 to 2015

*β*_*i*_, *γ*_*i*_, *δ*_*i*_, *ω*_*i*_, *φ*_*i*_ are coefficients for our different variables and *ε*_*it*_ error term

Health as human capital is captured through expenditure on health, the life expectancy of an individual at birth and the proportion of the population that makes up the labour force of the economy. This study also highlights the importance of trade to the economy: researchers hypothesised that with a healthy human capital, production will increase through higher productivity of labour, thus, when the business climate is favourable it can create higher value added to the goods and services produced. Household consumption is equally considered because a great proportion of household income is spent on consumption in developing countries and it in turn reflects the level of domestic demand which has a multiplier effect on industry value added and thus economic growth.

This study seeks to find evidence for causality between health expenditure and economic growth and to verify the existence of co-integration, thus long-run relationships between the variables of the study. It also seeks to verify if the impact of health expenditure on economic growth is higher in African countries that achieved the Abuja Accord than those of CEMAC countries that did not. The granger causality test, panel co-integration test and panel OLS, panel fully modified ordinary least squares (FMOLS) and panel dynamic ordinary least squares (DOLS) models are employed to corroborate results and avoid the problems inherent in one method. Unit root tests are used to check if a series is stationary or not. A series is stationary if its probability distribution does not change over time. The Augmented Dickey fuller (ADF) test developed by Im et al. [39] will be used in this study. The combined Im, Pesaran and Shin(IPS) and Fisher tests would be employed.

Testing for the existence of a long-run relationship between health expenditure and economic growth often require the use of the Johansen’s procedure. However, the power of the Johansen test in multivariate systems with small sample size can be severely distorted. Thus, it is imperative to combine information from time series as well as cross-section data. Panel co-integration tests are employed. Several tests were developed by Kao [40] and Pedroni [41, 42] to examine the existence of co-integration in a multivariate framework. Their proposed statistics test the null hypothesis of no co-integration versus the alternative of co-integration. Unfortunately, pooling time series has resulted in substantial losses as far as permissible heterogeneity of the individual time series are concerned. It is paramount that in the process of pooling time series as much heterogeneity as possible among individual time series should be maintained. The process of testing for co-integration among variables should permit as much heterogeneity among the individual countries of the panel as possible. If pooled results rely on homogeneous panel co-integration theory, then common slope coefficients are imposed. Pesaran and Smith [43] highlighted that if a common estimator is used due to differences among the individual countries, then health expenditure and economic growth are not cointegrated. Pedroni [41] residual test is used in this study, seven tests would be used, and four for within-dimension panel and three for between-dimension group, the within-dimension panel tests also highlights the weighted statistics.

The granger causality test will be used to verify the direction of causality between the variables of the study, with special interest being given to the direction of causality between health expenditure and economic growth. The granger causality is very sensitive with number of lags used. The test has four possible outcomes: a) neither variable Granger causes the other, b) unidirectional causality from x to y, c) unidirectional causality from y to x, d) both variables Granger cause each other.

Several methods can be used for estimation in a panel framework with co-integration, amongst which we have: OLS, Fully Modified OLS (FMOLS), dynamic OLS(DOLS), and Pooled Mean Group (PMG). Analysis of the properties (the finite sample proprieties of the OLS estimator, the t-statistic, the bias-corrected OLS estimator, and the bias-corrected t-statistic) of the OLS estimator by Chen et al. [44] analysed shows that the bias-corrected OLS estimator does not generally improve over the OLS estimator. Other alternatives such as the FMOLS estimator or the DOLS estimator can be more appropriate in co-integrated panel regressions. FMOLS is well known in conventional time series econometrics because it is believed to eliminate serial correlation in the errors and endogeneity in the regressors. However, Kao and Chiang [45] demonstrated that both the Fully Modified OLS (FMOLS) and OLS both show signs of small sample bias and that the dynamic OLS (DOLS) estimator can outperform both estimators. Three estimators with error correction will be considered in this paper: Panel OLS, Fully Modified OLS (FMOLS) and dynamic OLS (DOLS) to examine the relationship between health expenditure and economic growth empirically.

## Data

In order to achieve the research objectives for this paper, Time series data from 1995 to 2015 was extracted from the World Development Indicators [46] of the World Bank. Due to high out of pocket expenditures in developing countries, the [46]) data base may not capture fully this informal expenditure, however it is the most reliable source of data for this study. This is inspired by the work of Mandiefe and Tieguhong [33] that considered GDP per capita as an indicator of economic growth. Variables on health expenditure used in this study are health expenditure per capita (logged), and life expectancy. Household consumption per capita (logged), labour force (logged) and trade are the other control variables used in this study. All these variables were extracted from the World Development Indicators and analysed using Eviews version 8. The sample for this study is made up of six (06) CEMAC member states and five other Sub-Saharan African countries that achieve the Abuja declaration, this is rather a small sample compared to Countries in Africa, thus a generalisation may lead to faulty conclusions due to the small size of the sample. Table 2 shows the two set of countries that make up our sample.

**Table 2:** Countries that make up the sample

African Countries (GGHE/GGE more than 15%)	CEMAC Countries (GGHE/GGE less than 15%)
Botswana, Rwanda, Zambia Madagascar, Togo	Cameroon, Equatorial Guinea, Gabon, Chad, Central African Republic, Democratic republic of Congo

*GGE* General government expenditure, *GGHE* General government health expenditure

Source: Compiled by author

## Results and discussion

### Results of ADF test

We see from Table 3 below that at level with trend and intercept the variables are not stationary. This means that the properties (Mean, variance, autocorrelation) of the time series data is not constant, therefore it is imperative to differentiate and test for stationarity again. At first difference, our results show that the time series properties are now constant. We see that the properties for the time series data for CEMAC countries and other Sub-Saharan African countries that spend more than 15% of the general government expenditure on health are stationary after first difference. We therefore say they are integrated of order one. This result highlights that there is a high possibility of a long-run relationship between economic growth and health expenditure, the probability that these variables are co-integrated is high. In order to verify this, the panel Pendroni’s residual co-integration test is effectuated with results provided in Table 4.

**Table 3:** Augmented Dickey Fuller Unit root test

CEMAC	Augmented Dickey Fuller test				Decision
	Level		First Difference		
	Trend & inter	Probability	Trend & inter	Probability	
GDP per capita	11.1034	0.5201	30.7002	0.0022	I(1)
Health expenditure per capita	3.76014	0.9874	26.3409	0.0096	I(1)
Household final consumption expenditure per capita (constant 2010 US$)	7.45014	0.8265	50.1558	0.0000	I(1)
Labour force, total	16.3967	0.1737	32.0656	0.0014	I(1)
Life expectancy at birth, total (years)	3.34533	0.4564	92.7717	0.0000	I(1)
Trade (% of GDP)	5.45676	0.4567	34.4610	0.0002	I(1)
Other african countries (>15% GGE)					
GDP per capita	10.9147	0.3642	40.3050	0.0000	I(1)
Health expenditure per capita	9.44517	0.4904	35.7159	0.0001	I(1)
Household final consumption expenditure per capita (constant 2010 US$)	4.52449	0.9206	28.1659	0.0017	I(1)
Labour force, total	6.89184	0.7356	16.7841	0.0793	I(1)
Life expectancy at birth, total (years)	5.78906	0.6789	76.0157	0.0000	I(0)
Trade (% of GDP)	16.0930	0.0970	42.7828	0.0000	I(1)

Source: Computed by author

**Table 4:** Panel co-integration test results

Pedroni Residual Cointegration Test		CEMAC(HE < 15% GGE)		Other african countries(HE > 15% GGE)	
		Statistic	Weighted Statistic	Statistic	Weighted Statistic
Within-dimension (panel)	Panel v-Statistic	5.320171***	−1.934143	−0.079408*	−1.629980
	Panel rho-Statistic	1.101854	1.583020	1.390759	2.080915
	Panel PP-Statistic	−6.944674***	−3.660656***	−4.191776***	−1.824957**
	Panel ADF-Statistic	−1.735961**	−2.395129***	−3.924845***	−2.128257***
Between-dimension (group)	Group rho-Statistic	2.199777		2.590251	
	Group PP-Statistic	−4.895312***		−2.152590***	
	Group ADF-Statistic	−1.533469*		−2.641490***	

*GGE* General government expenditure, *GGHE* General government health expenditure, *HE* Health Expenditure

The test statistics are normalized so that the asymptotic distribution is standard normal. *, **, *** indicate rejection of the null hypothesis of non-co-integration at the 10, 5,and 1% significance levels, based respectively on critical values of 1.281, 1.644 and 2.326

Source: Computed by author

### Panel Co-integration results

We see from Table 3 that the variables are co-integrated, panel co-integration test developed by Pedroni [42] is employed to empirically verify if there is co-integration.

The results from the panel co-integration test for CEMAC countries shows that for the seven within-dimension and between-dimension tests with normal statistics, five are significant as for the weighted statistics, while of the four within dimension tests two are significant. As for the other Sub-Saharan countries respecting the Abuja target, five of the within-dimension and between-dimension statistics are significant while two of the weighted within-dimension statistics are significant. We can therefore conclude from these test statistics that there is co-integration between the variables. This means that co-integration panel regression is necessary.

### Results of the granger causality test

The objective of this test is to verify the direction of causality between the variables of our study. The null hypothesis of this test states that there is no granger causality between the variables while the alternative states that there is causality and it equally indicates if the causality is unidirectional or bidirectional. Table 5 below shows the results of the granger causality test for CEMAC region and other Sub-Saharan countries.

**Table 5:** Results of granger causality test

Null Hypothesis:	Other african countries (PHE > 15% GGE)			CEMAC (PHE < 15% GGE)		
	Obs	F-Statistic	Prob.	Obs	F-Statistic	Prob.
Health Expenditure does not Granger Cause LGDP	95	5.78615	0.0043	114	4.83873	0.0097
GDP per capita does not Granger Cause Health expenditure		2.04999	0.1347		13.5136	6.E-06
Household consumption does not Granger Cause GDP per capita	95	2.44313	0.0926	114	2.08910	0.1287
GDP per capita does not Granger Cause Household consumption		0.08120	0.9221		0.94095	0.3934
Labour force does not Granger Cause GDP per capita	95	3.76413	0.0269	114	6.57896	0.0020
GDP per capita does not Granger Cause Labour force		0.92280	0.4011		2.30750	0.1044
Life expectancy does not Granger Cause GDP per capita	95	0.93059	0.3981	114	0.10912	0.8967
GDP per capita does not Granger Cause Life expectancy		4.01096	0.0214		0.70521	0.4962
Trade does not Granger Cause GDP per capita	95	0.99131	0.3751	114	1.37170	0.2580
GDP per capita does not Granger Cause Trade		2.40805	0.0958		2.66348	0.0742
Household consumption does not Granger Cause Health expenditure	95	0.72830	0.4856	114	0.75915	0.4705
Health expenditure does not Granger Cause Household consumption		3.50142	0.0343		0.24121	0.7861
Labour force does not Granger Cause Health expenditure	95	4.42441	0.0147	114	11.4054	3.E-05
Health expenditure does not Granger Cause labour force		0.47943	0.6207		3.48352	0.0342
Life expectancy does not Granger Cause Health expenditure	95	1.05275	0.3532	114	0.38290	0.6828
Health expenditure does not Granger Cause Life expectancy		3.56348	0.0324		1.76211	0.1765
Trade does not Granger Cause health expenditure	95	1.15248	0.3205	114	1.58412	0.2098
Health expenditure does not Granger Cause Trade		0.29987	0.7417		0.39338	0.6757
Labour force does not Granger Cause Household consumption	95	3.07424	0.0511	114	1.31762	0.2720
Cause Household consumption does not Granger Cause labour force		0.39595	0.6742		1.63605	0.1995
Life expectancy does not Granger Cause Household consumption	95	1.79853	0.1714	114	0.97617	0.3800
Household consumption does not Granger Cause life expectancy		1.67952	0.1923		6.40920	0.0023
Trade does not Granger Cause Household consumption	95	1.76174	0.1776	114	2.22514	0.1129
Household consumption does not Granger Cause Trade		0.39271	0.6764		0.34313	0.7103
Life expectancy does not Granger Cause labour force	95	0.09075	0.9133	114	0.89907	0.4099
labour force does not Granger Cause life expectancy		8.89750	0.0003		8.63025	0.0003
Trade does not Granger Cause labour force	95	0.21222	0.8092	114	4.22751	0.0171
labour force does not Granger Cause trade		0.09455	0.9099		4.14838	0.0184
Trade does not Granger Cause life expectancy	95	1.88887	0.1572	114	2.75488	0.0680
Life expectancy does not Granger Cause Trade		1.16546	0.3164		0.01484	0.9853

Source: Computed by author

Our results show that for the other Sub-Saharan countries there is a unilateral causality running from GDP per capita to health expenditure, health expenditure does not granger cause GDP per capita. As for the CEMAC region there is bidirectional causality between GDP per capita and health expenditure. This means that the multiplier effect of health expenditure as GDP per capita increases in the CEMAC region is higher than that of the other countries that respect the Abuja target. This implies that, increasing expenditure on health can be a very important mechanism to increase the quality of human capital and thus economic growth, however this is not a direct mechanism where increase health expenditure will translate to economic growth, the necessary institutional framework have to be efficient and corrupt free to spur economic growth. The theoretical assumption is that economic growth and health are components of a feedback system or circle as health generates wealth and wealth generates health, but this feedback mechanism is not perfectly responsive. This is principally because other country specific proxies are important parameters to the responsiveness of increased health expenditure on economic growth. This explains the possibility of unilateral causality from economic growth to health expenditure for other sub-Saharan countries.

Our results equally show a unilateral relationship between labour force and GDP per capita, labour force granger cause GDP per capita but GDP per capita does not granger cause labour force. This holds for both the CEMAC region and the other five Sub-Saharan countries that achieve the Abuja target.

We see from above that the high expenditure on health in the five Sub-Saharan countries achieving the Abuja declaration have a unilateral relationship running from GDP per capita to life expectancy. However as for countries in the CEMAC region there is no relationship between GDP per capita and life expectancy. This means that countries respecting the Abuja declaration turn to enjoy faster economic growth because the quality of their human capital is improved by increased health expenditure.

Our granger causality test also highlights that health expenditure granger cause household consumption but household consumption does not granger cause health expenditure, this is true for the five Sub-Saharan countries that respect the Abuja target on health expenditure. There is no relationship between health expenditure and household expenditure for CEMAC member countries. We therefore see that when the government spends more on health, the quality of human capital is higher, thus productivity increases, income per head increases leading to increase in household consumption per head.

There is a bidirectional relationship between labour force and health expenditure for CEMAC countries but a unilateral relationship running from labour force to health expenditure for the five countries achieving the Abuja declaration running from labour force to health expenditure. We equally see a unilateral relationship running from health expenditure to life expectancy for the five countries achieving the Abuja declaration, thus any increase in health expenditure will effectively leads to increase in the standard of living and life expectancy of citizens in these countries, but this is not true for CEMAC member countries.

There is a unilateral relationship running from labour force to household consumption for both groups of countries while there is a bidirectional relationship between trade and labour force in CEMAC and no relationship for the five countries achieving the Abuja declaration. This highlights the importance of labour force for the growth of CEMAC economies, therefore, productivity would greatly be enhanced if there is appropriate investment in health infrastructure and personnel in this sub-region.

### Panel regression results

This paper makes use of three panel regression models: panel OLS, fully modified OLS (FMOLS), dynamic panel OLS(DOLS). The results can be seen from Table 6 below. The results shows that globally all the three models for both groups of countries have a very high explicative power with more than 96% of the variance of the dependent variable explained by the independent variables. This can be seen from the R-squared and the adjusted R square.

**Table 6:** Panel Regression results

GDP per capita(dependent Variable)	CEMAC (HE < 15% GGE)			Other african countries (>15% GGE)		
	Panel OLS	FMOLS	DOLS	Panel OLS	FMOLS	DOLS
Health expenditure per capita	0.304995*** (4.777252)	0.207370* (1.779484)	0.304995*** (3.269052)	0.128414*** (2.797812)	0.156037*** (2.515290)	0.381941*** (17.48346)
Household final consumption expenditure per capita (constant 2010 US$)	−0.046588 (−0.516395)	−0.220483 (−0.992283)	−0.046588 (−0.353367)	0.522384*** (7.736917)	0.478174*** (5.119678)	−0.727531*** (−28.95947)
Labour force, total	0.582692*** (2.816049)	0.670658* (1.894656)	0.582692* (1.927009)	0.165731* (1.798202)	0.090819 (0.648487)	1.379817*** (29.15916)
Life expectancy at birth, total (years)	−2.196447** (−2.587529)	−1.681589 (−1.084255)	−2.196447 (−1.770634)	0.654035*** (4.384715)	0.933523*** (3.748145)	−0.757376*** (−6.573151)
Trade (% of GDP)	0.476166*** (4.935038)	0.456469*** (3.087371)	0.476166*** (3.377024)	−0.242128*** (−2.867136)	−0.348765*** (2.994127)	−0.632140*** (−30.47444)
Constant	4.279689* (1.927313)			−0.842740 (−0.776672)		
R-squared	0.974961	0.979337	0.974961	0.987795	0.987782	0.999997
Adjusted R-squared	0.972784	0.972372	0.972784	0.986639	0.986561	0.999991
Long-run variance		0.076700	0.087998		0.012616	6.82E-07

Note: Figures in parentheses are t-stat. of the estimates; and “***”, “**”, and “*” indicates 1, 5, and 10% significant levels, respectively

Source: Computed by author

In this regard, it is imperative to invest in health. Drawing conclusions from the DOLS model because it is best suited for co-integrated panel regressions, we see that a unit change in health expenditure will lead to an increase in GDP per capita by 0.38% for the five Sub-Saharan African countries that respect the Abuja target while a unit change in health expenditure will lead to a 0.30% increase in GDP per capita for CEMAC countries. We see that achieving the Abuja targets leads to increase in GDP of these countries at a higher pace 0.08%than those of CEMAC countries who do not meet the target.

A negative relationship between household expenditure and GPD per capita is also noticed. It can be seen for countries of the CEMAC sub region and the five Sub-Saharan African countries that achieve the Abuja target; a unit increase in household consumption leads to a fall in GDP per capita. This relationship is significant for the five Sub-Saharan African countries that achieve the Abuja target, but not significant for countries of the CEMAC sub-region.

The results shows that labour force has a significant positive impact on GPD per capita for all the three models and for both regions with a higher effect for the five Sub-Saharan African countries that achieve the Abuja target and that invest in human capital resulting in a healthy labour force.

Life expectancy has a negative but not significant effect on GDP per capita for CEMAC countries and a negative and significant effect on GDP per capita for the five Sub-Saharan African countries that achieve the Abuja target. Trade expressed as a percentage of GDP has a positive and significant effect on GDP per capita for CEMAC countries and a negative and significant effect on GDP per capita for the five Sub-Saharan African countries that achieve the Abuja target.

## Discussion of results

The main results of this study shows that expenditure on health is co-integrated with economic growth, with a significant positive impact of expenditure on health for countries in the CEMAC sub region and the five other African countries that achieve the Abuja target. But the impact is higher for countries that achieve the Abuja target than CEMAC countries that do not achieve the target. These results confirm those of Baldacci et al. [22], who used a panel of 120 developing countries to show that health expenditure have a positive and significant impact on growth over a particular timeframe. He equally highlighted that health expenditure is a flow but not a stock. These results equally corroborates with that of Bloom et al. [23] who estimated a production function and concluded that there is a statistical significant positive effect of health expenditure on economic growth.

Evidence of co-integration between health expenditure and economic growth for both sets of studies goes in line with the works of Mandiefe and Tieguhong [33] who used the vector error correction model to verify if there is any short run or long run relationship between health expenditure and economic growth. Their results showed that there is no short-run relationship but there is a long-run relationship between the variables. These results are in contradiction to the works of Ogundipe et al. [31], who examined the impact of health expenditure on economic growth in Nigeria but noticed a negative and significant impact. The result of Ogundipe et al. [31] is equally contrary to that of Bakare and Sanmi [30] in Nigeria who came to a conclusion that expenditure on health have a positive and significant effect on economic growth.

The long-run relationship between health expenditures and other proxies of health like life expectancy and economic growth in this study corroborates with that of Dreiger and Reimers [29] who used panel co-intergration regression on a sample of 15 OECD countries to conclude that there is evidence of long-run relationship between economic growth and health expenditure and other proxies of health. Mila and Sadeghi verified the relationship between expenditure on health and economic growth by examining the direction of causality and if there is any long-run or short run relationship between health expenditure and economic growth. The results showed a short-run causality running from GDP to health expenditure, but there is a bilateral long-run relationship between economic growth and health expenditure. These findings confirm the results of our study.

Contemporary debate of how life expectancy affects economic growth is expanded in this paper, divergent views and empirical results have emerged relative to this topic; Barro [47] used a sample of 84 countries to show that there is a positive and significant impact of life expectancy on economic growth. Bloom et al. [48] used a sample of 104 countries and concluded that increase in life expectancy leads to increase in GDP by 2.65 to 4%. These authors highlight that life expectancy have a positive impact on GDP per capita. The results of this study however shows a negative and significant impact of life expectancy on economic growth, this view is supported by other empirical studies like that of Acemoglu and Johnson [49] who showed that health care inovations increases population growth and life expectancy which instead reduces per capital GDP per head. Barro and Lee [50] used new income data set to show a negative and significant impact of life expectancy on economic growth.

The important role of human capital in every economy shows that health care expenditure assures good quality labour force which is translated by increase in economic output thus increase in economic growth. The regression results shows a positive and significant impact of labour force on economic growth, Thus economies with young and qualified labour force turn to stir faster economic growth. This result contradicts results from developed countries with ageing labour force life that of Canada, with a negative impact of labour force on economic growth [51]. Empirical investigations in developing countries reveal a positive relationship between labour force participation and economic growth [52].

The positive and significant impact of health expenditure on economic growth in this study confirms empirical works of Hashmati [15]; Bakare and Sanmi [30]; Aguayo-Rico and Iris [28]; Dreger and Reimers [29] who confirm a positive and significant relationship between health expenditure and economic growth. The result from this paper and relate studies contradicts that of [53, 54], who studied a sample of 49 African countries from 1996 to 2010 to verify the impact of expenditure on education and health on economic growth. Their results showed that expenditures on education and health have a negative impact on economic growth.

## Conclusion

This paper reviews the application of the Abuja declaration of 2001, 15 years after signatories to this declaration accepted to allocate 15% of government expenditure on health. As of 2013 only five African countries had more than 15% of government expenditure allocated to the health sector. This paper compares these five countries with countries the CEMAC region where no country meets the required target. Co-integration tests, granger causality and panel data co-integration analysis were conducted to reveal interesting results.

Results from the study shows that there is co-integration between health expenditures, proxies of health and economic growth; therefore there is a long-run relationship between the variables of our study for both CEMAC countries and the five other countries that achieved the Abuja declaration. Bi-directional causality between economic growth and health expenditure was noticed for CEMAC countries while countries achieving the Abuja declaration portrayed a unilateral causality running from economic growth to health expenditure. This therefore means that income is an important element in explaining healthcare expenditure, thus increase in general level of income can stimulate increase in health expenditure [55]. This highlights the potential of the impact of a healthy labour force for CEMAC region on economic growth.

It is important to acknowledge that pumping money into the health sector does not automatically lead to economic growth, other accommodating conditions have to be in place for optimal response of health expenditure. Governance related issues should assure that health investments are directed to improving health standard of the population. Economic growth may increase by other country specific policies, not necessarily health expenditure, however this study underscores the importance of efficient health expenditure as a potential driver of economic growth. Potential positive benefits can quickly be felt when the governance framework of countries are transparent in financial management.

Indeed, from the results of this study, people who live in countries that spend more than 15% of their government expenditure turn to live longer than those living in countries of the CEMAC region that spend less than 15% of their government expenditure on the health sector. Therefore, it could be argued that higher expenditures on health may partly explain some reductions in the cases of untimely deaths due to lack of appropriate medical care. Increase in health expenditure leads to higher economic growth from this study, but this increase is not proportionate for CEMAC countries as compared to the five other African countries that achieve the Abuja declaration. Countries that achieve this declaration enjoy a higher increase in economic growth when a unit of expenditure on health is added than the CEMAC countries. Therefore a unit increase in expenditure on health would can potentially lead to an additional 0.08% increase in economic growth for countries that increase expenditure on health especially when they take necessary conditions to meet intended development objectives.

### Policy implication

This study outlines the importance of achieving the Abuja declaration and goes further to extend literature on the possible positive impact of increasing health expenditure on economic growth. From the results acquired, the following policy implications are pertinent for African countries.

This study shows that health expenditure is a fundamental determinant of economic growth of every nation and that increasing expenditure on health leads to higher growth rates. African countries should endeavor to meet and surpass the target of the Abuja declaration of 2001. One possible measure that could be taken to raise funds to meet this target could be by increasing taxes on products such as cigarettes and other products of ostentation and rechanneling the extra revenue generated to investment in healthcare. One of the important drawbacks to funding of Sub-Saharan countries is poor governance, thus measures to assure a fluid target based expenditure is imperative. In countries where poor governance is alarming, as the World Bank states, increasing public spending both from external donors and the government does not necessarily lead to the desired development outcomes [11]. Performance based financing can be an important mechanism that potential donors and government agencies can use. It is also believed to increase transparency and accountability in achieving targets [56]. Meessen et al. [56] also argues that it improves the allocative efficiency of resources especially in low-income developing countries where resources are quite limited. Thus an efficient financing mechanism with greater emphasis on the processes leading to the performance goal.
